# P-1330. Evaluation of Multi-Dose Strategy of Phage K and Vancomycin Delivery to Methicillin-Resistant Staphylococcus epidermidis (MRSE) Biofilms on Orthopedic Screws

**DOI:** 10.1093/ofid/ofaf695.1518

**Published:** 2026-01-11

**Authors:** Guyu Li, Melissa J Karau, Sebastian Herren, Robin Patel

**Affiliations:** Mayo Clinic, Rochester, MN; Mayo Clinic, Rochester, MN; Mayo Clinic, Rochester, MN; Mayo Clinic, Rochester, MN

## Abstract

**Background:**

Phage therapy has emerged as a generally safe approach, as demonstrated in preclinical and clinical studies. However, data on efficacy, including optimal dosing frequency, remain limited. MRSE is a leading cause of foreign body-associated osteomyelitis and a potential target for phage therapy. Here, the activity of phage K alone and in combination with vancomycin (VAN), administered once (QD) or three times (TID) daily, against biofilms formed by two orthopedic infection-associated MRSE isolates on orthopedic screws was evaluated.

**Fig 1.**
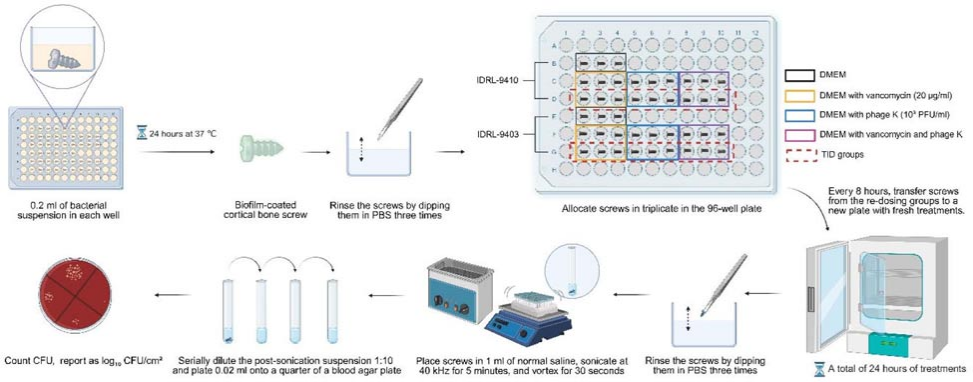
in vitro multi-dose assay of methicillin-resistant S. epidermidis biofilm-coated cortical bone screws. IDRL, Infectious Diseases Research Laboratory; DMEM, Dulbecco's Modified Eagle Medium; PBS, phosphate-buffered saline; CFU, colony-forming units. Created in https://BioRender.com

**Fig 2.**
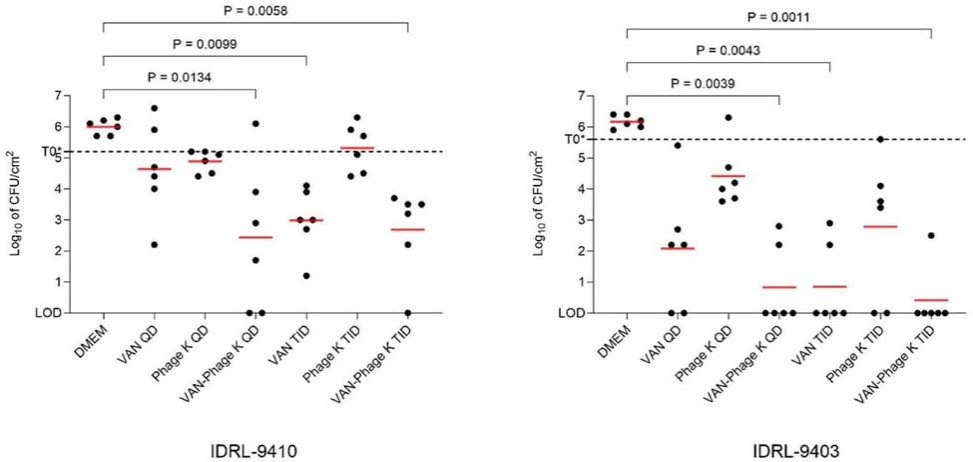
Quantities of biofilm on screws after 24 hours of treatment. *Log10 CFU/cm² of biofilm on screws before treatments. Biofilm burden of the MRSE isolates prior to treatment ranged from 5.7 to 6.3 log_10_ CFU/cm². A Kruskal-Wallis test was performed to evaluate the statistical significance of biofilm reduction between treatment groups. The minimum inhibitory concentration (MIC), minimum biofilm inhibitory concentration (MBIC), and minimum biofilm bactericidal concentration (MBBC) of vancomycin for both IDRL-9410 and IDRL-9403 were 2, 2, and >128 µg/mL, respectively.

**Methods:**

MRSE IDRL-9410 and IDRL-9403 biofilms were formed on titanium cortical bone screws (D 1.5 mm, L 4 mm, surface area 0.33 cm²) in 0.2 mL of tryptic soy broth containing 10⁶ CFU/mL (Fig 1); testing was performed in triplicate on two separate days. After 24 hours, screws were rinsed with PBS and randomly assigned to: Dulbecco’s Modified Eagle Medium (DMEM); vancomycin (VAN, 20 μg/mL) in DMEM; phage K (10⁹ PFU/mL) in DMEM; or VAN and phage K in DMEM. Every 8 hours, screws from the TID groups were transferred to new plates containing fresh treatment, with QD groups left in the original material. 24 hours post-treatment, screws were quantitatively cultured. Bactericidal activity was defined as ≥ 3 log₁₀ CFU/cm² reduction compared to the DMEM group, and synergy as ≥ 2 log₁₀ CFU/cm² reduction in the combination group compared to VAN or phage alone.

**Results:**

After 24 hours, bactericidal activity was observed for QD and TID VAN-phage K groups against IDRL-9410 (p < 0.05 and p < 0.01, respectively) and IDRL-9403 (both p < 0.01). Synergy was observed in the VAN-phage K QD group, with a 2.2 log₁₀ CFU/cm² reduction for IDRL-9410 compared to VAN alone (Fig 2). 3.0 and 5.3 log₁₀ CFU/cm² reductions were observed in the VAN TID groups for IDRL-9410 and IDRL-9403, respectively, compared to the DMEM group (both p < 0.01). There was no statistically significant difference in biofilm reduction between the QD and TID groups for phage K alone or VAN-phage K for either strain.

**Conclusion:**

Combination treatment with phage K and VAN, administered QD or TID, showed greater biofilm reduction compared to phage K alone. Increasing phage dosing frequency did not enhance biofilm reduction under the conditions studied.

**Disclosures:**

Robin Patel, MD, a patent on Bordetella pertussis/parapertussis PCR issued, a patent on a device/method for sonication, a patent on PET imaging of bacterial infection: a patent on Bordetella pertussis/parapertussis PCR issued, a patent on a device/method for sonication, a patent on PET imaging of bacterial infection|MicuRx Pharmaceuticals and bioMérieux: Grant/Research Support|PhAST, Day Zero Diagnostics, DEEPULL DIAGNOSTICS, S.L., Nostics, HealthTrackRx, bioMérieux and CARB-X: Advisor/Consultant|Up-to-Date and the Infectious Diseases Board Review Course: Honoraria

